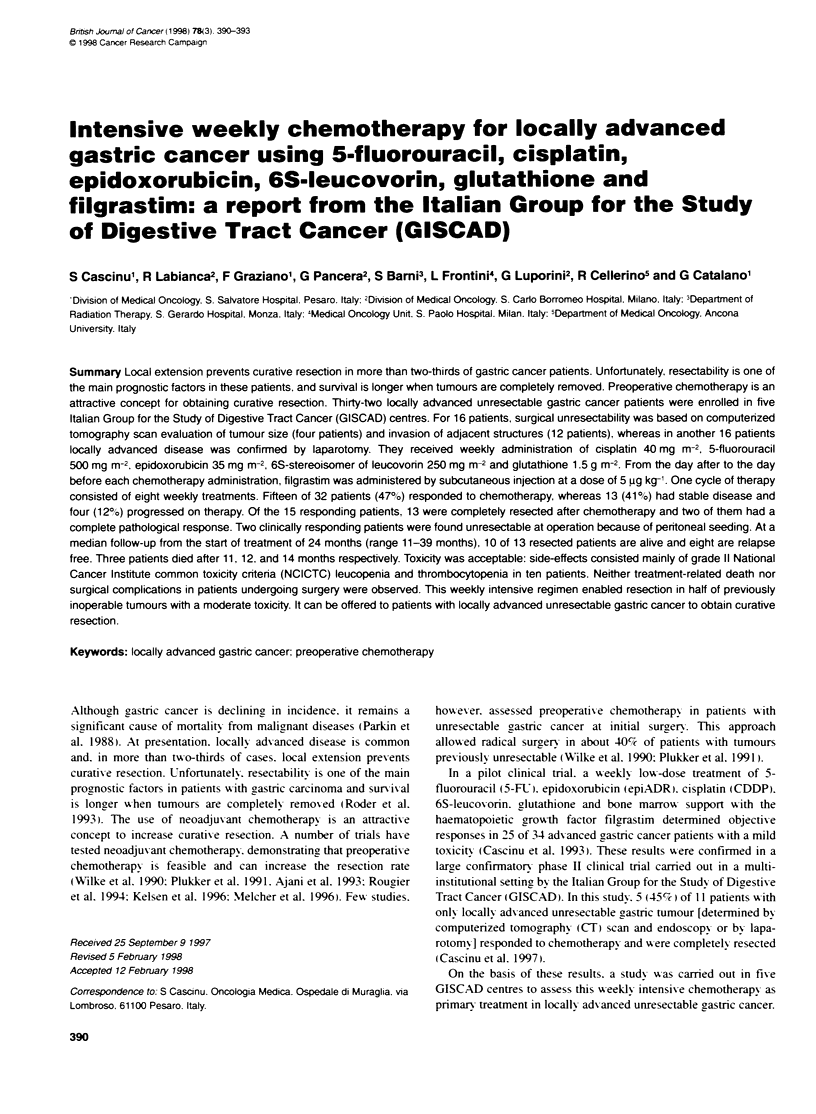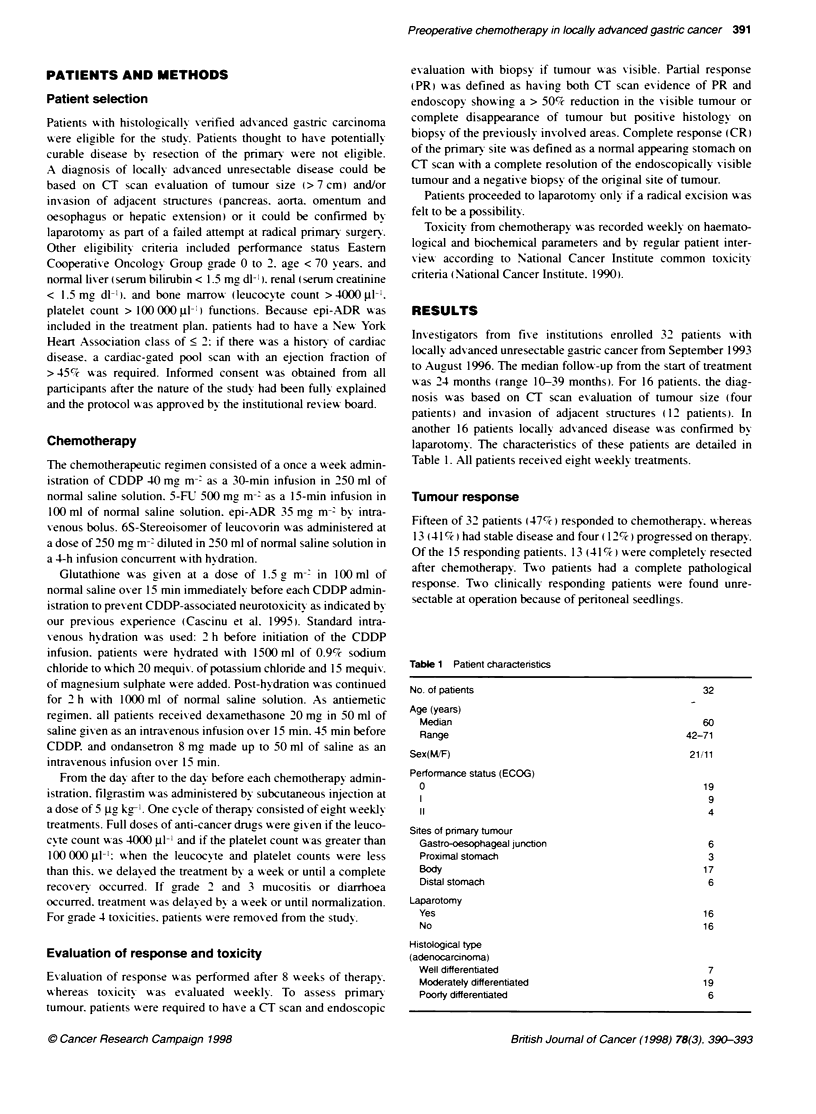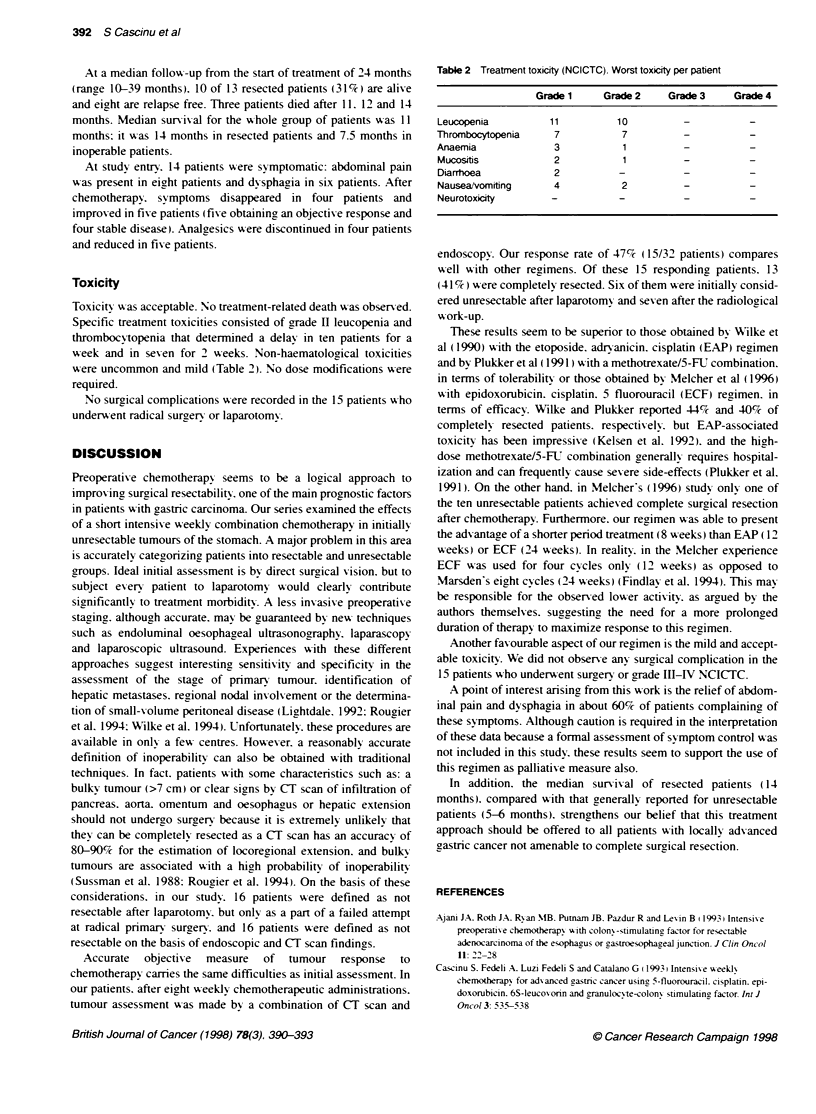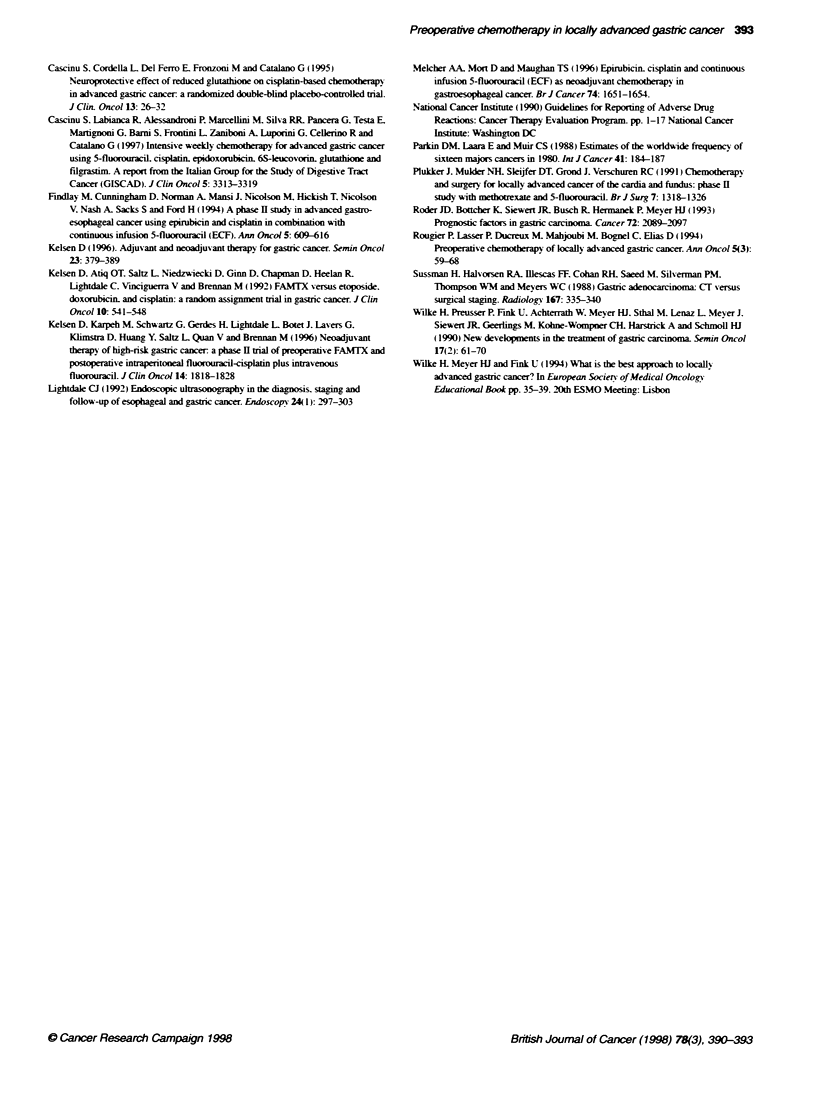# Intensive weekly chemotherapy for locally advanced gastric cancer using 5-fluorouracil, cisplatin, epidoxorubicin, 6S-leucovorin, glutathione and filgrastim: a report from the Italian Group for the Study of Digestive Tract Cancer (GISCAD).

**DOI:** 10.1038/bjc.1998.505

**Published:** 1998-08

**Authors:** S. Cascinu, R. Labianca, F. Graziano, G. Pancera, S. Barni, L. Frontini, G. Luporini, R. Cellerino, G. Catalano

**Affiliations:** Division of Medical Oncology, S. Salvatore Hospital, Pesaro, Italy.

## Abstract

Local extension prevents curative resection in more than two-thirds of gastric cancer patients. Unfortunately, resectability is one of the main prognostic factors in these patients, and survival is longer when tumours are completely removed. Preoperative chemotherapy is an attractive concept for obtaining curative resection. Thirty-two locally advanced unresectable gastric cancer patients were enrolled in five Italian Group for the Study of Digestive Tract Cancer (GISCAD) centres. For 16 patients, surgical unresectability was based on computerized tomography scan evaluation of tumour size (four patients) and invasion of adjacent structures (12 patients), whereas in another 16 patients locally advanced disease was confirmed by laparotomy. They received weekly administration of cisplatin 40 mg m(-2), 5-fluorouracil 500 mg m(-2), epidoxorubicin 35 mg m(-2), 6S-stereoisomer of leucovorin 250 mg m(-2) and glutathione 1.5 g m(-2). From the day after to the day before each chemotherapy administration, filgrastim was administered by subcutaneous injection at a dose of 5 microg kg(-1). One cycle of therapy consisted of eight weekly treatments. Fifteen of 32 patients (47%) responded to chemotherapy, whereas 13 (41 %) had stable disease and four (12%) progressed on therapy. Of the 15 responding patients, 13 were completely resected after chemotherapy and two of them had a complete pathological response. Two clinically responding patients were found unresectable at operation because of peritoneal seeding. At a median follow-up from the start of treatment of 24 months (range 11-39 months), 10 of 13 resected patients are alive and eight are relapse free. Three patients died after 11, 12, and 14 months respectively. Toxicity was acceptable: side-effects consisted mainly of grade II National Cancer Institute common toxicity criteria (NCICTC) leucopenia and thrombocytopenia in ten patients. Neither treatment-related death nor surgical complications in patients undergoing surgery were observed. This weekly intensive regimen enabled resection in half of previously inoperable tumours with a moderate toxicity. It can be offered to patients with locally advanced unresectable gastric cancer to obtain curative resection.


British Journal of Cancer (1998) 78(3), 390–393
© 1998 Cancer Research Campaign

### Intensive weekly chemotherapy for locally advanced gastric cancer using 5-fluorouracil, cisplatin, epidoxorubicin, 6S-leucovorin, glutathione and filgrastim: a report from the Italian Group for the Study of Digestive Tract Cancer (GISCAD)

**S Cascinu[1], R Labianca[2], F Graziano[1], G Pancera[2], S Barni[3], L Frontini[4], G Luporini[2], R Cellerino[5] and G Catalano[1]**

[1]Division of Medical Oncology, S. Salvatore Hospital, Pesaro, Italy; [2]Division of Medical Oncology, S. Carlo Borromeo Hospital, Milano, Italy; [3]Department of Radiation Therapy, S. Gerardo Hospital, Monza, Italy; [4]Medical Oncology Unit, S. Paolo Hospital, Milan, Italy; [5]Department of Medical Oncology, Ancona University, Italy

**Summary** Local extension prevents curative resection in more than two-thirds of gastric cancer patients. Unfortunately, resectability is one of the main prognostic factors in these patients, and survival is longer when tumours are completely removed. Preoperative chemotherapy is an attractive concept for obtaining curative resection. Thirty-two locally advanced unresectable gastric cancer patients were enrolled in five Italian Group for the Study of Digestive Tract Cancer (GISCAD) centres. For 16 patients, surgical unresectability was based on computerized tomography scan evaluation of tumour size (four patients) and invasion of adjacent structures (12 patients), whereas in another 16 patients locally advanced disease was confirmed by laparotomy. They received weekly administration of cisplatin 40 mg $m^{-2}$, 5-fluorouracil 500 mg $m^{-2}$, epidoxorubicin 35 mg $m^{-2}$, 6S-stereoisomer of leucovorin 250 mg $m^{-2}$ and glutathione 1.5 g $m^{-2}$. From the day after to the day before each chemotherapy administration, filgrastim was administered by subcutaneous injection at a dose of 5 μg $kg^{-1}$. One cycle of therapy consisted of eight weekly treatments. Fifteen of 32 patients (47%) responded to chemotherapy, whereas 13 (41%) had stable disease and four (12%) progressed on therapy. Of the 15 responding patients, 13 were completely resected after chemotherapy and two of them had a complete pathological response. Two clinically responding patients were found unresectable at operation because of peritoneal seeding. At a median follow-up from the start of treatment of 24 months (range 11–39 months), 10 of 13 resected patients are alive and eight are relapse free. Three patients died after 11, 12, and 14 months respectively. Toxicity was acceptable: side-effects consisted mainly of grade II National Cancer Institute common toxicity criteria (NCICTC) leucopenia and thrombocytopenia in ten patients. Neither treatment-related death nor surgical complications in patients undergoing surgery were observed. This weekly intensive regimen enabled resection in half of previously inoperable tumours with a moderate toxicity. It can be offered to patients with locally advanced unresectable gastric cancer to obtain curative resection.

**Keywords:** locally advanced gastric cancer; preoperative chemotherapy

Although gastric cancer is declining in incidence, it remains a significant cause of mortality from malignant diseases (Parkin et al, 1988). At presentation, locally advanced disease is common and, in more than two-thirds of cases, local extension prevents curative resection. Unfortunately, resectability is one of the main prognostic factors in patients with gastric carcinoma and survival is longer when tumours are completely removed (Roder et al, 1993). The use of neoadjuvant chemotherapy is an attractive concept to increase curative resection. A number of trials have tested neoadjuvant chemotherapy, demonstrating that preoperative chemotherapy is feasible and can increase the resection rate (Wilke et al, 1990; Plukker et al, 1991, Ajani et al, 1993; Rougier et al, 1994; Kelsen et al, 1996; Melcher et al, 1996). Few studies, however, assessed preoperative chemotherapy in patients with unresectable gastric cancer at initial surgery. This approach allowed radical surgery in about 40% of patients with tumours previously unresectable (Wilke et al, 1990; Plukker et al, 1991).

In a pilot clinical trial, a weekly low-dose treatment of 5-fluorouracil (5-FU), epidoxorubicin (epiADR), cisplatin (CDDP), 6S-leucovorin, glutathione and bone marrow support with the haematopoietic growth factor filgrastim determined objective responses in 25 of 34 advanced gastric cancer patients with a mild toxicity (Cascinu et al, 1993). These results were confirmed in a large confirmatory phase II clinical trial carried out in a multi-institutional setting by the Italian Group for the Study of Digestive Tract Cancer (GISCAD). In this study, 5 (45%) of 11 patients with only locally advanced unresectable gastric tumour [determined by computerized tomography (CT) scan and endoscopy or by laparotomy] responded to chemotherapy and were completely resected (Cascinu et al, 1997).

On the basis of these results, a study was carried out in five GISCAD centres to assess this weekly intensive chemotherapy as primary treatment in locally advanced unresectable gastric cancer.

*Received 25 September 9 1997*
*Revised 5 February 1998*
*Accepted 12 February 1998*

*Correspondence to:* S Cascinu, Oncologia Medica, Ospedale di Muraglia, via Lombroso, 61100 Pesaro, Italy.

### PATIENTS AND METHODS

### Patient selection

Patients with histologically verified advanced gastric carcinoma were eligible for the study. Patients thought to have potentially curable disease by resection of the primary were not eligible. A diagnosis of locally advanced unresectable disease could be based on CT scan evaluation of tumour size (> 7 cm) and/or invasion of adjacent structures (pancreas, aorta, omentum and oesophagus or hepatic extension) or it could be confirmed by laparotomy as part of a failed attempt at radical primary surgery. Other eligibility criteria included performance status Eastern Cooperative Oncology Group grade 0 to 2, age < 70 years, and normal liver (serum bilirubin < 1.5 mg $dl^{-1}$), renal (serum creatinine < 1.5 mg $dl^{-1}$), and bone marrow (leucocyte count > 4000 $\mu l^{-1}$, platelet count > 100 000 $\mu l^{-1}$) functions. Because epi-ADR was included in the treatment plan, patients had to have a New York Heart Association class of ≤ 2; if there was a history of cardiac disease, a cardiac-gated pool scan with an ejection fraction of > 45% was required. Informed consent was obtained from all participants after the nature of the study had been fully explained and the protocol was approved by the institutional review board.

### Chemotherapy

The chemotherapeutic regimen consisted of a once a week administration of CDDP 40 mg $m^{-2}$ as a 30-min infusion in 250 ml of normal saline solution, 5-FU 500 mg $m^{-2}$ as a 15-min infusion in 100 ml of normal saline solution, epi-ADR 35 mg $m^{-2}$ by intravenous bolus. 6S-Stereoisomer of leucovorin was administered at a dose of 250 mg $m^{-2}$ diluted in 250 ml of normal saline solution in a 4-h infusion concurrent with hydration.

Glutathione was given at a dose of 1.5 g $m^{-2}$ in 100 ml of normal saline over 15 min immediately before each CDDP administration to prevent CDDP-associated neurotoxicity as indicated by our previous experience (Cascinu et al, 1995). Standard intravenous hydration was used: 2 h before initiation of the CDDP infusion, patients were hydrated with 1500 ml of 0.9% sodium chloride to which 20 mequiv. of potassium chloride and 15 mequiv. of magnesium sulphate were added. Post-hydration was continued for 2 h with 1000 ml of normal saline solution. As antiemetic regimen, all patients received dexamethasone 20 mg in 50 ml of saline given as an intravenous infusion over 15 min, 45 min before CDDP, and ondansetron 8 mg made up to 50 ml of saline as an intravenous infusion over 15 min.

From the day after to the day before each chemotherapy administration, filgrastim was administered by subcutaneous injection at a dose of 5 $\mu g\ kg^{-1}$. One cycle of therapy consisted of eight weekly treatments. Full doses of anti-cancer drugs were given if the leucocyte count was 4000 $\mu l^{-1}$ and if the platelet count was greater than 100 000 $\mu l^{-1}$; when the leucocyte and platelet counts were less than this, we delayed the treatment by a week or until a complete recovery occurred. If grade 2 and 3 mucositis or diarrhoea occurred, treatment was delayed by a week or until normalization. For grade 4 toxicities, patients were removed from the study.

### Evaluation of response and toxicity

Evaluation of response was performed after 8 weeks of therapy, whereas toxicity was evaluated weekly. To assess primary tumour, patients were required to have a CT scan and endoscopic evaluation with biopsy if tumour was visible. Partial response (PR) was defined as having both CT scan evidence of PR and endoscopy showing a > 50% reduction in the visible tumour or complete disappearance of tumour but positive histology on biopsy of the previously involved areas. Complete response (CR) of the primary site was defined as a normal appearing stomach on CT scan with a complete resolution of the endoscopically visible tumour and a negative biopsy of the original site of tumour.

Patients proceeded to laparotomy only if a radical excision was felt to be a possibility.

Toxicity from chemotherapy was recorded weekly on haematological and biochemical parameters and by regular patient interview according to National Cancer Institute common toxicity criteria (National Cancer Institute, 1990).

### RESULTS

Investigators from five institutions enrolled 32 patients with locally advanced unresectable gastric cancer from September 1993 to August 1996. The median follow-up from the start of treatment was 24 months (range 10–39 months). For 16 patients, the diagnosis was based on CT scan evaluation of tumour size (four patients) and invasion of adjacent structures (12 patients). In another 16 patients locally advanced disease was confirmed by laparotomy. The characteristics of these patients are detailed in Table 1. All patients received eight weekly treatments.

### Tumour response

Fifteen of 32 patients (47%) responded to chemotherapy, whereas 13 (41%) had stable disease and four (12%) progressed on therapy. Of the 15 responding patients, 13 (41%) were completely resected after chemotherapy. Two patients had a complete pathological response. Two clinically responding patients were found unresectable at operation because of peritoneal seedlings.

**Table 1** Patient characteristics

| | |
|---|---|
| No. of patients | 32 |
| Age (years) | |
| Median | 60 |
| Range | 42–71 |
| Sex(M/F) | 21/11 |
| Performance status (ECOG) | |
| 0 | 19 |
| I | 9 |
| II | 4 |
| Sites of primary tumour | |
| Gastro-oesophageal junction | 6 |
| Proximal stomach | 3 |
| Body | 17 |
| Distal stomach | 6 |
| Laparotomy | |
| Yes | 16 |
| No | 16 |
| Histological type (adenocarcinoma) | |
| Well differentiated | 7 |
| Moderately differentiated | 19 |
| Poorly differentiated | 6 |

© Cancer Research Campaign 1998

At a median follow-up from the start of treatment of 24 months (range 10–39 months), 10 of 13 resected patients (31%) are alive and eight are relapse free. Three patients died after 11, 12 and 14 months. Median survival for the whole group of patients was 11 months; it was 14 months in resected patients and 7.5 months in inoperable patients.

At study entry, 14 patients were symptomatic: abdominal pain was present in eight patients and dysphagia in six patients. After chemotherapy, symptoms disappeared in four patients and improved in five patients (five obtaining an objective response and four stable disease). Analgesics were discontinued in four patients and reduced in five patients.

### Toxicity

Toxicity was acceptable. No treatment-related death was observed. Specific treatment toxicities consisted of grade II leucopenia and thrombocytopenia that determined a delay in ten patients for a week and in seven for 2 weeks. Non-haematological toxicities were uncommon and mild (Table 2). No dose modifications were required.

No surgical complications were recorded in the 15 patients who underwent radical surgery or laparotomy.

### DISCUSSION

Preoperative chemotherapy seems to be a logical approach to improving surgical resectability, one of the main prognostic factors in patients with gastric carcinoma. Our series examined the effects of a short intensive weekly combination chemotherapy in initially unresectable tumours of the stomach. A major problem in this area is accurately categorizing patients into resectable and unresectable groups. Ideal initial assessment is by direct surgical vision, but to subject every patient to laparotomy would clearly contribute significantly to treatment morbidity. A less invasive preoperative staging, although accurate, may be guaranteed by new techniques such as endoluminal oesophageal ultrasonography, laparascopy and laparoscopic ultrasound. Experiences with these different approaches suggest interesting sensitivity and specificity in the assessment of the stage of primary tumour, identification of hepatic metastases, regional nodal involvement or the determination of small-volume peritoneal disease (Lightdale, 1992; Rougier et al, 1994; Wilke et al, 1994). Unfortunately, these procedures are available in only a few centres. However, a reasonably accurate definition of inoperability can also be obtained with traditional techniques. In fact, patients with some characteristics such as: a bulky tumour (>7 cm) or clear signs by CT scan of infiltration of pancreas, aorta, omentum and oesophagus or hepatic extension should not undergo surgery because it is extremely unlikely that they can be completely resected as a CT scan has an accuracy of 80–90% for the estimation of locoregional extension, and bulky tumours are associated with a high probability of inoperability (Sussman et al, 1988; Rougier et al, 1994). On the basis of these considerations, in our study, 16 patients were defined as not resectable after laparotomy, but only as a part of a failed attempt at radical primary surgery, and 16 patients were defined as not resectable on the basis of endoscopic and CT scan findings.

Accurate objective measure of tumour response to chemotherapy carries the same difficulties as initial assessment. In our patients, after eight weekly chemotherapeutic administrations, tumour assessment was made by a combination of CT scan and endoscopy. Our response rate of 47% (15/32 patients) compares well with other regimens. Of these 15 responding patients, 13 (41%) were completely resected. Six of them were initially considered unresectable after laparotomy and seven after the radiological work-up.

These results seem to be superior to those obtained by Wilke et al (1990) with the etoposide, adryanicin, cisplatin (EAP) regimen and by Plukker et al (1991) with a methotrexate/5-FU combination, in terms of tolerability or those obtained by Melcher et al (1996) with epidoxorubicin, cisplatin, 5 fluorouracil (ECF) regimen, in terms of efficacy. Wilke and Plukker reported 44% and 40% of completely resected patients, respectively, but EAP-associated toxicity has been impressive (Kelsen et al, 1992), and the high-dose methotrexate/5-FU combination generally requires hospitalization and can frequently cause severe side-effects (Plukker et al, 1991). On the other hand, in Melcher's (1996) study only one of the ten unresectable patients achieved complete surgical resection after chemotherapy. Furthermore, our regimen was able to present the advantage of a shorter period treatment (8 weeks) than EAP (12 weeks) or ECF (24 weeks). In reality, in the Melcher experience ECF was used for four cycles only (12 weeks) as opposed to Marsden's eight cycles (24 weeks) (Findlay et al, 1994). This may be responsible for the observed lower activity, as argued by the authors themselves, suggesting the need for a more prolonged duration of therapy to maximize response to this regimen.

Another favourable aspect of our regimen is the mild and acceptable toxicity. We did not observe any surgical complication in the 15 patients who underwent surgery or grade III–IV NCICTC.

A point of interest arising from this work is the relief of abdominal pain and dysphagia in about 60% of patients complaining of these symptoms. Although caution is required in the interpretation of these data because a formal assessment of symptom control was not included in this study, these results seem to support the use of this regimen as palliative measure also.

In addition, the median survival of resected patients (14 months), compared with that generally reported for unresectable patients (5–6 months), strengthens our belief that this treatment approach should be offered to all patients with locally advanced gastric cancer not amenable to complete surgical resection.

**Table 2** Treatment toxicity (NCICTC). Worst toxicity per patient

| | Grade 1 | Grade 2 | Grade 3 | Grade 4 |
|---|---|---|---|---|
| Leucopenia | 11 | 10 | – | – |
| Thrombocytopenia | 7 | 7 | – | – |
| Anaemia | 3 | 1 | – | – |
| Mucositis | 2 | 1 | – | – |
| Diarrhoea | 2 | – | – | – |
| Nausea/vomiting | 4 | 2 | – | – |
| Neurotoxicity | – | – | – | – |

### REFERENCES

Ajani JA, Roth JA, Ryan MB, Putnam JB, Pazdur R and Levin B (1993) Intensive preoperative chemotherapy with colony-stimulating factor for resectable adenocarcinoma of the esophagus or gastroesophageal junction. *J Clin Oncol* **11**: 22–28

Cascinu S, Fedeli A, Luzi Fedeli S and Catalano G (1993) Intensive weekly chemotherapy for advanced gastric cancer using 5-fluorouracil, cisplatin, epidoxorubicin, 6S-leucovorin and granulocyte-colony stimulating factor. *Int J Oncol* **3**: 535–538

© Cancer Research Campaign 1998

Cascinu S. Cordella L. Del Ferro E. Fronzoni M and Catalano G (1995) Neuroprotective effect of reduced glutathione on cisplatin-based chemotherapy in advanced gastric cancer: a randomized double-blind placebo-controlled trial. *J Clin. Oncol* **13**: 26–32

Cascinu S. Labianca R. Alessandroni P. Marcellini M. Silva RR. Pancera G. Testa E. Martignoni G. Barni S. Frontini L. Zaniboni A. Luporini G. Cellerino R and Catalano G (1997) Intensive weekly chemotherapy for advanced gastric cancer using 5-fluorouracil. cisplatin. epidoxorubicin. 6S-leucovorin. glutathione and filgrastim. A report from the Italian Group for the Study of Digestive Tract Cancer (GISCAD). *J Clin Oncol* **5**: 3313–3319

Findlay M. Cunningham D. Norman A. Mansi J. Nicolson M. Hickish T. Nicolson V. Nash A. Sacks S and Ford H (1994) A phase II study in advanced gastro-esophageal cancer using epirubicin and cisplatin in combination with continuous infusion 5-fluorouracil (ECF). *Ann Oncol* **5**: 609–616

Kelsen D (1996). Adjuvant and neoadjuvant therapy for gastric cancer. *Semin Oncol* **23**: 379–389

Kelsen D. Atiq OT. Saltz L. Niedzwiecki D. Ginn D. Chapman D. Heelan R. Lightdale C. Vinciguerra V and Brennan M (1992) FAMTX versus etoposide. doxorubicin. and cisplatin: a random assignment trial in gastric cancer. *J Clin Oncol* **10**: 541–548

Kelsen D. Karpeh M. Schwartz G. Gerdes H. Lightdale L. Botet J. Lavers G. Klimstra D. Huang Y. Saltz L. Quan V and Brennan M (1996) Neoadjuvant therapy of high-risk gastric cancer: a phase II trial of preoperative FAMTX and postoperative intraperitoneal fluorouracil-cisplatin plus intravenous fluorouracil. *J Clin Oncol* **14**: 1818–1828

Lightdale CJ (1992) Endoscopic ultrasonography in the diagnosis. staging and follow-up of esophageal and gastric cancer. *Endoscopy* **24**(1): 297–303

Melcher AA. Mort D and Maughan TS (1996) Epirubicin. cisplatin and continuous infusion 5-fluorouracil (ECF) as neoadjuvant chemotherapy in gastroesophageal cancer. *Br J Cancer* **74**: 1651–1654.

National Cancer Institute (1990) Guidelines for Reporting of Adverse Drug Reactions: Cancer Therapy Evaluation Program. pp. 1–17 National Cancer Institute: Washington DC

Parkin DM. Laara E and Muir CS (1988) Estimates of the worldwide frequency of sixteen majors cancers in 1980. *Int J Cancer* **41**: 184–187

Plukker J. Mulder NH. Sleijfer DT. Grond J. Verschuren RC (1991) Chemotherapy and surgery for locally advanced cancer of the cardia and fundus: phase II study with methotrexate and 5-fluorouracil. *Br J Surg* **7**: 1318–1326

Roder JD. Bottcher K. Siewert JR. Busch R. Hermanek P. Meyer HJ (1993) Prognostic factors in gastric carcinoma. *Cancer* **72**: 2089–2097

Rougier P. Lasser P. Ducreux M. Mahjoubi M. Bognel C. Elias D (1994) Preoperative chemotherapy of locally advanced gastric cancer. *Ann Oncol* **5**(**3**): 59–68

Sussman H. Halvorsen RA. Illescas FF. Cohan RH. Saeed M. Silverman PM. Thompson WM and Meyers WC (1988) Gastric adenocarcinoma: CT versus surgical staging. *Radiology* **167**: 335–340

Wilke H. Preusser P. Fink U. Achterrath W. Meyer HJ. Sthal M. Lenaz L. Meyer J. Siewert JR. Geerlings M. Kohne-Wompner CH. Harstrick A and Schmoll HJ (1990) New developments in the treatment of gastric carcinoma. *Semin Oncol* **17**(2): 61–70

Wilke H. Meyer HJ and Fink U (1994) What is the best approach to locally advanced gastric cancer? In *European Society of Medical Oncology Educational Book* pp. 35–39. 20th ESMO Meeting: Lisbon

© Cancer Research Campaign 1998